# Bone Marrow Findings in Renal Patients: A Single Renal Specialist Center Experience

**DOI:** 10.7759/cureus.18912

**Published:** 2021-10-19

**Authors:** Kiran Nasir, Ruqaya Qureshi, Hina Qureshi, Murtaza Dhrolia, Aasim Ahmad

**Affiliations:** 1 Nephrology, The Kidney Centre Post Graduate Training Institute, Karachi, PAK; 2 Hematology, The Kidney Centre Post Graduate Training Institute, Karachi, PAK; 3 Nephrology, The Kidney Center Post Graduate Training Institute, Karachi, PAK

**Keywords:** maintenance hemodialysis, chronic kidney disease (ckd), hematological abnormalities, bone marrow biopsy (bmb), bone marrow aspirate

## Abstract

Objective

This study evaluated the importance of bone marrow aspiration and trephine biopsy (BM) for the diagnosis of underlying hematological abnormalities in renal patients.

Methods

This cross-sectional study on BM was carried out between August 2010 and April 2019, in our specialist renal center for various unexplained hematological abnormalities in patients with renal diseases [chronic kidney disease (CKD), end-stage renal disease (ESRD) requiring maintenance hemodialysis (MHD), patients with normal renal function but other nephrology and urology issues like stone disease and nephrotic syndrome].

Results

Out of 176 reported BM examinations, 48 (27.3%) were done on ESRD patients on MHD (CKD-D), and 69 (39.2%) on CKD patients not on MHD (CKD-nD). Fifty-nine (33.5%) BM were done on patients with normal renal function (n-CKD). The indication for BM was pancytopenia 50 (28.4%), unexplained anemia 39 (22.2%), and unexplained thrombocytopenia 43 (24.4%). In 91 (51.7%) patients BM was normal. In 30 (17%) patients multiple myeloma (MM) was diagnosed on BM, out of which 18 (26.1%), nine (18%), three (5.3%) were CKD-nD, CKD-D, and n-CKD patients, respectively. In 11 (6.3%) patients BM was suggestive of myelodysplasia (MD), out of these 11 patients, five (10%) were CKD-D patients.

Conclusion

BM is an underutilized method of diagnosis of hematological abnormalities in renal patients. Our study revealed the importance of BM examination, especially in patients with CKD.

## Introduction

Chronic kidney disease (CKD) is growing as a public health burden especially in third world countries like Pakistan. Lack of healthcare education, scarce or unequal distribution of health care services, increasing prevalence of diabetes mellitus and hypertension, and lower socioeconomic status are the major reason for the increasing prevalence of CKD. The prevalence of CKD is variable in different countries. It is 4.3-16.7% in USA [1], 17.3% in Germany [2], 6.7-18.3% in China [3], 0.7-17.2% in India [4], and 5.0-31.2% in Pakistan [5,6].

CKD is associated with various hematological changes including anemia, prolonged bleeding time, platelet and white blood cell dysfunction, pancytopenia, thrombocytopenia, and coagulopathies [7]. Anemia leads to greater cardiovascular workload, left ventricular hypertrophy, and increased mortality in CKD patients. Thrombocytopenia, platelet dysfunction, and coagulopathies increase the risk of bleeding, while abnormalities in the function of white blood cells increase the risk of infection in these patients.

Bone marrow aspiration and trephine biopsy (BM) can be used to diagnose various hematological abnormalities especially in patients where the obvious cause is not found. It can help as a tool to diagnose diseases like lymphoproliferative disorders, multiple myeloma (MM), idiopathic thrombocytopenic purpura (ITP), myelodysplasia (MD), and infections like tuberculosis [8]. It can also help in the evaluation of iron stores [9] as well as causes of erythropoietin resistance like pure red cell aplasia (PRCA) [10] or hyperparathyroidism [11].

BM is an underutilized tool for the diagnosis of various hematological disorders in patients with CKD. We reviewed all BM done at our specialist renal (nephrology, urology, and renal transplant) center in patients who presented to us with unexplained hematological findings and underwent BM.

## Materials and methods

We did a cross-sectional audit of all BM done between August 2010 and April 2019 at our center, The Kidney Centre Post Graduate Training Institute, Karachi, Pakistan, after exemption from the Ethics Review Committee (Ref. No. 76-NEPH-042019). Patients were divided into three groups: CKD patients not on MHD (CKD-nD), patients on MHD (CKD-D), and patients with normal renal function (n-CKD). As we did an audit of all BM reports, we also included n-CKD patients who visited our center for various nephrology and urology issues (stone disease, nephrotic syndrome). BM requested from other hospitals with scarce clinical data, done at our center, were excluded. All BM reports were included regardless of patient’s age, gender and ethnicity. Demographic data (age, sex, comorbid conditions like diabetes mellitus, hypertension, malignancy, hypothyroidism, ischemic heart disease), laboratory results (complete blood counts, reticulocyte count, iron studies, hepatitis B and C profile), indication and results of BM were collected.

According to WHO, anemia is defined as hemoglobin (Hb) <13 g/dL in males and <12 g/dL in females. The normal range of white blood cell (WBC) count is between 4.0-11.0 x 10^9^/L, and a result below 4.0 x 10^9^/L is considered as low and above 11 x 10^9^/L is considered as high. Thrombocytopenia was defined as platelet count < 100 x 10^3^/uL. Iron stores on BM were divided into six grades (Grade 0: No stainable iron; Grade 1: Small iron particles just visible in reticulum cells using an oil objective; Grade 2: Small, Sparse iron particles in reticulum cells, visible at lower power; Grade 3: Numerous small particles in reticulum cells; Grade 4: Larger particles with a tendency to aggregate into clumps; Grade 5: Dense, Large clumps; Grade 6: Very large clumps and extracellular iron) [12].

Blood counts were performed using a fully automated hematology analyzer Sysmex Xs1000i (Sysmex Corporation, Kobe, Japan). BM aspiration and trephine biopsies are routinely carried out using a 16-gauge lumbar puncture needle and an 11-gauge disposable bone marrow biopsy needle respectively.

BM aspiration and trephine biopsy was obtained from the posterior iliac crest after taking written informed consent, following standard guidelines. Smears from BM aspirates were stained using Leishman stain for morphology whereas, smears with spicules were assessed for hemosiderin using Perl stain. When it was difficult to obtain a good aspirate, trephine touch imprints were taken to complement the trephine biopsy reporting. Bone cores were processed and then routinely stained with hematoxylin and eosin (H&E) for routine microscopy. Other stains like reticulin and periodic acid-Schiff (PAS), Sudan black B (SBB), and/or Congo red stain were used if required. All slides were examined under a standard clinical microscope on X10, X40, and X100 lenses. Polarized microscopy was used if smears were stained with Congo red. Frequencies with percentages were obtained for categorical data using SPSS Software version 21 (IBM Corp., Armonk, NY).

## Results

A total of 176 patients were included in our study in which the female to male ratio was 1:1.35, mean age was 49±18.7 years. Hypertension was the most common comorbid condition 50 (28.4%) in our patients while diabetes mellitus was present in 46 (26.1%) patients. Out of 176 patients, 69 (39.2%) were CKD-nD, 48 (27.3%) were CKD-D, and 59 (33.5%) were n-CKD. Table 1 describes the clinical and demographic characteristics of the patients.

**Table 1:** Demographic and clinical characteristics of patients N = Number of patients

Characteristics			N (%)
Gender	Male		101 (57.4)
	Female		75 (42.6)
Diabetes Mellitus			46 (26.1)
Hypertension			50 (28.4)
Ischemic Heart Disease			17 (9.7)
Hypothyroidism			3 (1.7)
Malignancy (Renal cell carcinoma)			1 (0.6)
Status of renal disease		n-CKD	59 (33.5)
		CKD-nD	69 (39.2)
		CKD-D	48 (27.3)

The most frequent indication for BM was pancytopenia 50 (28.4%). BM was done for persistently unexplained thrombocytopenia and unexplained anemia in 43 (24.4%) and 39 (22.2%) patients, respectively (Table 2). BM findings in the majority of our patients showed active erythropoiesis 108 (61.4%) and active granulopoiesis 155 (88.1%). Iron grading predominantly revealed Grade 4 iron stores in 60 (34.1%) patients while in 27 (15.3%) patients no iron stores were found.

**Table 2:** Indication for bone marrow examination N = Number of patients

Indications	N (%)
Pancytopenia	50 (28.4)
Thrombocytopenia	43 (24.4)
Anemia	39 (22.2)
Anemia and Thrombocytopenia	19 (10.8)
Leukocytosis	13 (2.3)
Leukopenia and Thrombocytopenia	6 (3.4)
Polycythemia	4 (2.3)
Anemia and Leukopenia	2 (1.1)

Most of the BM examined were normal in our study 91 (51.7%). In 30 (17%) patients, BM revealed MM; out of which 18 (26.1%), nine (18%), and three (5.3%) were CKD-nD, CKD-D, and n-CKD patients respectively. BM of 11 (6.3%) patients suggested MD, out of which five (10%) were CKD-D patients (Table 3).

**Table 3:** Bone Marrow findings according to the renal status of patients N = Number of patients; BM = Bone marrow aspiration and trephine biopsy; AML = Acute myelocytic leukemia; ALL = Acute lymphocytic leukemia; ITP = Idiopathic thrombocytopenic purpura; CLL = Chronic lymphocytic leukemia; MD = Myelodysplasia

Bone marrow findings		Status of renal disease N (%)			
		n-CKD	CKD-nD	CKD-D	Total
Erythropoiesis	Dyserythropoisis	14 (24.6)	19 (27.5)	20 (40)	53 (30.1)
	Active	40 (70.2)	45 (65.2)	23 (46)	108 (61.4)
	Increased	3 (5.3)	5 (7.2)	7 (14)	15 (8.5)
Granulopoiesis	Suppressed	2 (3.5)	4 (5.8)	7 (14)	13 (7.4)
	Active	55 (96.5)	60 (87)	40 (80)	155 (88.1)
	Increased	0	5 (7.2)	3 (6)	8 (4.5)
Megakaryocytes	Suppressed	2 (3.5)	9 (13)	6 (12)	17 (9.7)
	Adequate	55 (96.5)	60 (87)	44 (88)	159 (90.3)
Blast cells	Normal	55 (96.5)	61 (88.4)	50 (100)	166 (94.3)
	Increased	2 (3.5)	8 (11.6)	0	10 (5.7)
Trilineage Hematopoiesis	Reduced	20 (35.1)	23 (33.3)	14 (28)	57 (32.4)
	Adequate	32 (56.1)	36 (38.3)	26 (52)	94 (53.4)
	Increased	5 (8.8)	10 (14.5)	10 (20)	25 (14.2)
Iron Grading	Absent	11 (19.3)	10 (14.5)	6 (12)	27 (15.3)
	Grade 1	4 (7)	2 (2.9)	1 (20)	7 (4)
	Grade 2	13 (22.8)	8 (11.6)	3 (6)	24 (13.6)
	Grade 3	8 (14)	11 (15.9)	7 (14)	26 (14.8)
	Grade 4	19 (33.3)	26 (37.7)	15 (30)	60 (34.1)
	Grade 5	2 (3.5)	9 (13)	5 (10)	16 (9.1)
	Grade 6	0	3 (4.3)	13 (26)	16 (9.1)
Findings on BM examination	Normal	35 (61.4)	35 (50.7)	21 (42)	91 (51.7)
	Multiple myeloma	3 (5.3)	18 (26.1)	9 (18)	30 (17)
	Lymphoma	0	1 (1.4)	0	1 (0.6)
	AML	0	2 (2.9)	0	2 (1.1)
	ALL	1 (1.8)	0	0	1 (0.6)
	Iron deficiency	7 (12.3)	1 (1.4)	0	8 (4.5)
	Megaloblastic anemia	1 (1.8)	3 (4.3)	2 (4)	6 (3.4)
	ITP	1 (1.8)	0	0	1 (0.6)
	Aplastic anemia	1 (1.8)	3 (4.3)	2 (4)	6 (3.4)
	CLL	0	2 (2.9)	0	2 (1.1)
	Defective hemoglobinization	3 (5.3)	2 (2.9)	10 (20)	15 (8.5)
	MD	4 (7)	2 (2.9)	5 (10)	11 (6.3)
	Polycythemia rubra Vera	1 (1.8)	0	1 (2)	2 (1.1)

## Discussion

We evaluated all BM done in our specialist renal center to assess the importance of BM as a supportive under-utilized test for diagnosis of underlying hematological abnormalities in renal patients. CKD is associated with various hematological abnormalities, but like the general population, this particular cohort can have other hematological disorders like MM and leukemia. In our study, the most common indication for BM was unexplained pancytopenia in 50 (28.4%) patients.

BM was done for unexplained anemia in 39 (22.2%) of our patients. Anemia in CKD can be multifactorial. Factors include reduced production of erythropoietin (EPO), an absolute iron deficiency due to blood loss, impaired iron absorption, ineffective use of iron stores due to increased hepcidin level, systemic inflammation, reduced bone marrow response to EPO, reduced red cell life span, and deficiency of vitamin B12 and folic acid [13]. BM can be used as a tool to diagnose the underlying cause of unexplained anemia in CKD patients. Diagnosis and correction of anemia are important as chronic anemia predisposes them to high morbidity and mortality, mostly secondary to cardiovascular diseases [14]. The risk of left ventricular hypertrophy (LVH) and congestive heart failure (CHF) increases with anemia in CKD patients [15]. Unexplained anemia was the most common indication of BM in a study done by Weng et al. to assess the importance of BM pathology as a predictor of mortality in CKD patients on hemodialysis [16].

BM can be done in CKD patients with unexplained cytopenias including pancytopenia, to rule out underlying diseases such as granulomatous infections, hematological malignancies, or MD, which could be suggestive of underlying myelodysplastic syndrome (MDS) [17]. MD can present as unexplained erythropoietin-resistant anemia in CKD patients. In our study, BM of 11 (6.3%) patients suggested MD, out of which five (10%) were CKD-D patients.

We found Grade 4 iron stores in 60 (34.1%) patients, indicating adequate iron replacement therapy. As serum ferritin can be high in CKD patients secondary to inflammation, serum ferritin is often used with transferrin saturation (Tsat) to assess iron status, diagnose iron deficiency, and predict response to iron supplementation [18]. Mean serum ferritin was 619.6±654.2 and Tsat was 28.4±19.4, which correlates with BM iron staining in our patients.

Another important finding of our study was the diagnosis of MM on BM in 30 (17%) patients. MM is a plasma cell dyscrasia that accounts for almost 10% of all hematologic malignancies [19]. Patients with MM have approximately a 50% chance of developing acute kidney injury (AKI) or CKD during the course of their disease [20], while 25-50% have kidney dysfunction at presentation, and approximately 9% require hemodialysis (HD) [21]. Renal disease in MM range from renal insufficiency, proteinuria, renal tubular dysfunction, and rarely, Fanconi syndrome [22]. Estimation of plasma cells in BM is an important step in the diagnosis and monitoring of the course of MM. Early diagnosis of MM is important for better outcomes. Other than MM in our study population, other malignancies were also diagnosed including lymphoma, acute myelocytic leukemia (AML), chronic lymphocytic leukemia (CLL). As our center is a specialist renal center, we refer these patients to a clinical hematologist for further management.

Our study has provided the importance of BM in CKD patients. It is important for the nephrologists, as many times changes in blood cell lines like persistent anemia, thrombocytopenia, and pancytopenia are labelled as secondary to CKD and not investigated further leading to missed or late diagnosis of underlying hematological conditions like MM, which are treatable if diagnosed on time. Our study has certain limitations. This is a cross-sectional audit of BM reported at our specialist renal center and for this reason, we were unable to assess the progress and outcome of BM findings in these patients.

## Conclusions

CKD is a rapidly growing public health burden with increased morbidity and mortality. It is associated with various hematologic abnormalities that affect erythropoiesis, granulopoiesis, platelet function, coagulation, and the immune system. Erythropoietin deficiency causes anemia of chronic disease in CKD while presence of uremic toxins causes dysfunction of white blood cells and platelets. Despite that patients with CKD can develop other hematological abnormalities like MM, MDS, and lymphoma. BM is quiet helpful in diagnosis of these hematological diseases, which can be treated successfully if diagnosed on time. Our study showed importance of BM in CKD patients. We conclude that BM is an important and underutilized bedside procedure for the diagnosis of various underlying conditions that can cause anemia, and/or erythropoietin resistance, thrombocytopenia and pancytopenia in patients with CKD.
